# The impact of real-time 3d imaging by ultra-high speed optical coherence tomography in urothelial carcinoma

**DOI:** 10.1186/1471-2490-13-65

**Published:** 2013-12-01

**Authors:** Masaomi Ikeda, Kazumasa Matsumoto, Donghak Choi, Morihiro Nishi, Tetsuo Fujita, Kohji Ohbayashi, Kimiya Shimizu, Masatsugu Iwamura

**Affiliations:** 1 Department of Urology, Kitasato University School of Medicine, Kanagawa, Japan; 2 Center for Natural Science, Kitasato University, Kanagawa, Japan; 3 Graduate School of Medical Science, Kitasato University, Kanagawa, Japan; 4 Department of Ophthalmology, Kitasato University School of Medicine, Kanagawa, Japan

**Keywords:** Optical coherence tomography, Real-time 3D imaging, Urothelial carcinoma, Carcinoma in situ, Real-time optical biopsy

## Abstract

**Background:**

Optical coherence tomography (OCT) has become a promising diagnostic tool in many medical fields. In particular, noninvasive real-time optical biopsy of internal organs is one of the most attractive applications of OCT, enabling in situ diagnosis of carcinoma at an early stage. We used an ultra-high speed OCT system for real-time three-dimensional (3D) imaging of three excised specimens of advanced urothelial carcinoma (UC) and investigated the association of the images with results from histopathological examination.

**Case presentations:**

Case 1 was a 69-year-old man underwent radical cystectomy for muscle-invasive UC (pT2). Case 2 was a 53-year-old man underwent laparoscopic nephroureterectomy and partial cystectomy for left ureter carcinoma (pT2) and case 3 was a 77-year-old woman underwent radical cystectomy for advanced bladder carcinoma (pT3b). Real-time 3D OCT images of normal bladder wall and ureter showed three layers, including the urothelium, lamina propria, and muscularis layer. In contrast, normal structure was not seen in the muscle-invasive UC area or the scar tissue area.

**Conclusions:**

This study highlighted a new diagnostic method with potential application for UC diagnosis. We will investigate more cases in the future and expect improvement in the diagnosing efficiency of carcinoma in situ or organ-confined muscle-invasive cancer by cystoscopy or ureteroscopy with ultra-high speed OCT.

## Background

An estimated 386,300 new cases of urothelial carcinoma (UC) of the bladder occurred in 2008, during which the disease accounted for 150,200 deaths worldwide [1]. Nonmuscle-invasive UC accounts for more than 70% of newly diagnosed cases [2] and includes carcinoma in situ (CIS), a flat, high-grade, noninvasive intraurothelial carcinoma with a high risk of aggressive progression. Early detection and treatment of recurrent disease are required to maximize bladder preservation and patient survival [3]. However, early diagnosis of CIS, which is crucial for appropriate treatment, remains a clinical challenge. Conventional imaging methods, such as x-ray, computerized tomography (CT) and magnetic resonance imaging (MRI), fail to detect early UC due to their limited resolution. Although urinary cytology, fluorescence in situ hybridization and bladder tumor antigen test are highly sensitive screening techniques for the clinical detection of CIS and high grade UC, they cannot identify carcinoma locations to effectively guide transurethral resection (TUR) and treatment [4]. Cystoscopy, the direct visualization of the bladder surface via optical fibre bundles, is the current gold standard for the diagnosis of UC. In addition, narrow band imaging (NBI) cystoscopy can increase bladder cancer visualization and detection at the time of TUR [5-8]. However, cystoscopy sometimes misses flat lesions such as CIS, which may be visually normal. Therefore, conclusive diagnosis and staging of malignancy in patients with positive cytology relies on random biopsy, which may miss almost 50% of early flat UC [9].

Optical coherence tomography (OCT) is an emerging technology that provides noninvasive, real-time high-resolution (10 to 20 μm) imaging of the bladder wall in cross-section, showing the urothelium, lamina propria, and muscularis layer to a depth of about 2 mm. OCT for medical diagnostics was first reported by Huang et al. [10], and it has become a promising diagnostic tool for, among others, retinal diseases in ophthalmology, esophageal and pancreatico-biliary diseases in gastroenterology, and cutaneous diseases in dermatology. Noninvasive real-time optical biopsy of internal organs is one of the most attractive applications of OCT enabling in situ diagnosis of carcinoma at an early stage.

Recently, Ohbayashi et al. [11] and Choi et al. [12] reported an ultra-fast data processing system installed in an ultra-high speed OCT system that enables real-time display of various three-dimensional (3D) images without the limitation of diagnostic time. We used this new system to investigate real-time 3D imaging of excised tissue from three patients with advanced UC and compared images to results from histopathological examination.

### Case presentations

### OCT measurement

We reported OCT measurement previously [11,12]. Briefly, all the cases were measured by ultra-high speed OCT system immediately after radical cystectomy or nephroureterectomy. The OCT imaging area was demonstrated around 12 mm from the probe, and the depth is approximately 4 mm. We investigated images consisting mainly of the three areas such as cancerous, normal and boundary areas, and compared the histopathological examination of the same area.

### Case 1

A 69-year-old man underwent radical cystectomy for muscle-invasive UC (pT2). Histopathological examination revealed no evidence of malignancy (pT0) or scar tissue after TUR of the bladder. OCT images of normal bladder wall were characterized by three layers: a thin, dark upper layer representing the urothelium and a second bright layer corresponding to the lamina propria, followed by the thick, dark muscularis layer. In contrast, the three characteristic layers were not found in the scar tissue area. There was no thin, dark upper layer like the urothelium. The two layers that were observed, a bright thick layer and a dark thick layer, were revealed using OCT. OCT showed no residual tumorous lesion in the bladder wall.

### Case 2

**Figure 1:**
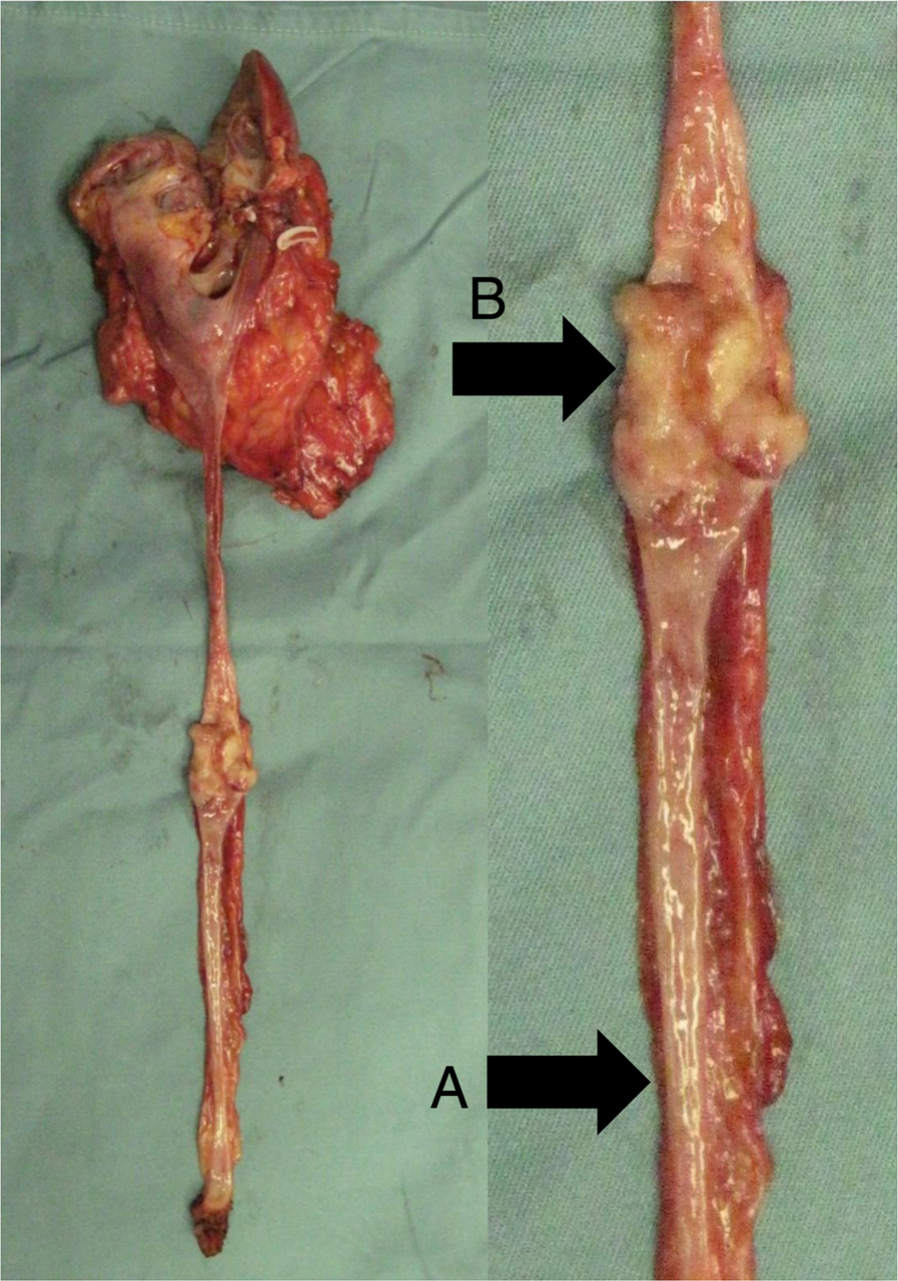
**Excised specimen of left ureter carcinoma (pT2a). (A)** Normal ureter wall. **(B)** Left ureter carcinoma.

**Figure 2:**
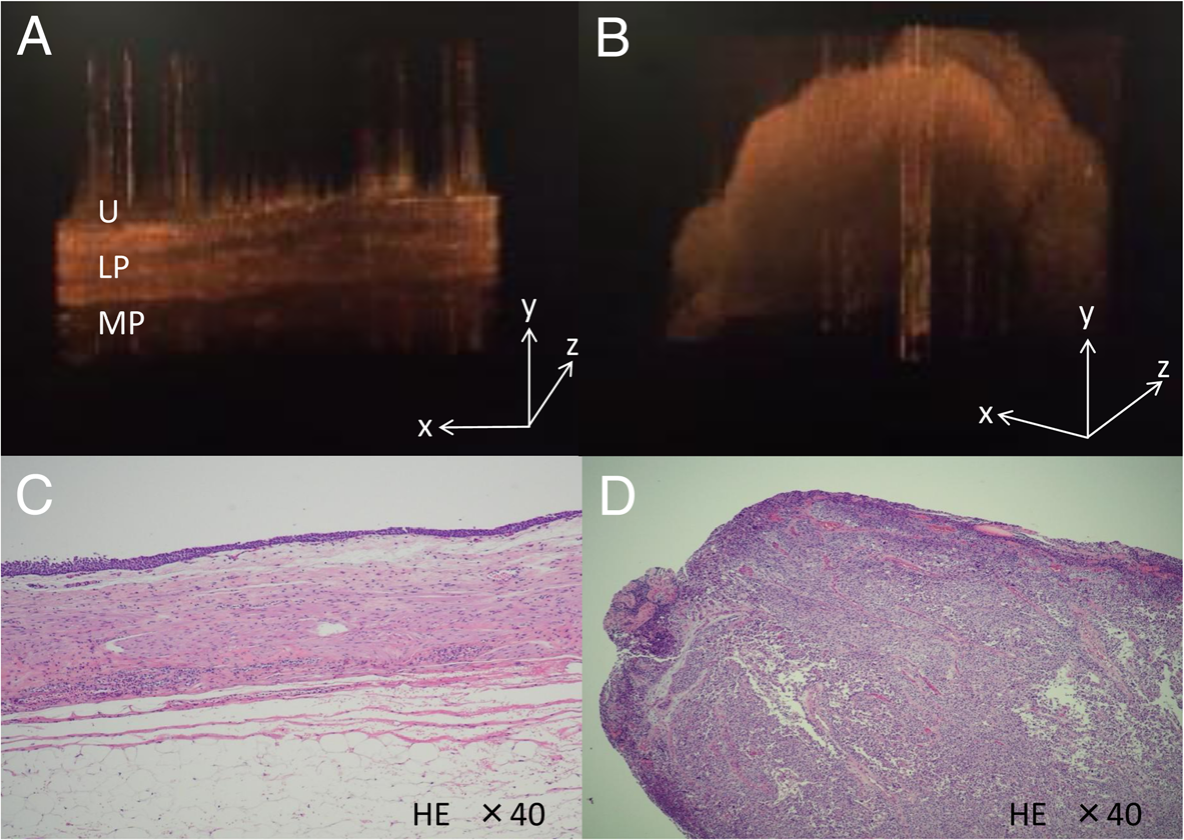
**Real-time 3D imaging by ultra-high speed OCT and histopathological examination of normal ureter wall and muscle-invasive urothelial carcinoma. (A)** A representative real-time 3D OCT image of normal ureter wall was characterized by three layers: a thin, dark upper layer for the urothelium (U) and a second bright layer corresponding to the lamina propria (LP), followed by the thick, dark muscularis layer (MP). **(B)** There were no layer structures in muscle-invasive ureter carcinoma area. **(C, D)** Histopathological images corresponding to **(A)** and **(B)**, respectively.

A 53-year-old man underwent laparoscopic nephroureterectomy and partial cystectomy for left ureter carcinoma (Figure 1). Histopathological examination revealed UC, pT2, grade 2 > 3. OCT images from a normal part of the ureter confirmed three layers, including the urothelium, lamina propria, and muscularis layer (Figure 2A, C). A single layer was seen in the advanced ureter carcinoma area (Figure 2B, D), and there were no normal structures.

### Case 3

A 77-year-old woman underwent radical cystectomy for advanced bladder carcinoma. Histopathological examination revealed small cell carcinoma, pT3b. Similar to the other cases, there were no distinguishable structural layers in OCT images of the muscle-invasive bladder cancer. Muscle-invasive lesions showed complete obliteration of any defined layers of the bladder wall.

## Discussion

TUR followed by histopathological examination is currently the standard for assessing depth of tumor penetration, which is a key determinant in treatment of bladder cancer. Pathologic stage is one of the strongest prognostic indicators of patient outcome [13]. However, specimen sampling is often limited for tissue analyses using TUR because of insufficient tissue collection, degeneration from coagulation and heterogeneous distribution of CIS in the bladder wall. In addition, conventional cystoscopy provides limited information regarding changes in the bladder wall, particularly below the surface. Therefore, when performing biopsy or resection, an urologist is guided only by the visual appearance of the tumor. Experienced urologists are able to differentiate benign or malignant lesions visually, but their ability to estimate grade and stage with cystoscopy alone is less accurate [14]. Overcoming these clinical limitations, NBI has been shown to increase the detection rate. NBI is an optical enhancement technology that consists of two bandwidths of illumination centred on 415 nm (blue) and 540 nm (green) that can increase the contrast between vasculature and superficial tissue structures of the mucosa [15]. Randomized prospective studies [5,6] and retrospective studies [7,8] have shown that NBI-assisted TUR could significantly reduce the residual tumor and recurrence risk. However, the NBI system is not suited to assessing the depth of tumor penetration. While conventional methods of imaging, including CT and MRI, have been useful for detecting advanced disease, they have done poorly in identifying nonmuscle-invasive cancer, particularly for CIS, or muscle-invasive cancer like T2a. This ultra-high speed OCT system may detect CIS that is not observed structural changes of submucosa in cystoscopy. In addition, it may diagnose the depth of the tumor penetration which cannot be seen in NBI. We believe that both detection of tumor and assessing the depth of tumor penetration by real-time 3D OCT can aid current modalities and lead to performing a precious diagnosis before the surgery in the future.

OCT provides unique information about underlying tissue microstructure that can augment diagnosis and improve staging of bladder cancer. The feasibility of OCT to delineate normal and abnormal bladder tissue has been shown in previous studies [16,17], and OCT has demonstrated excellent high sensitivity and specificity. Manyak et al. [16] evaluated 87 areas of interest; 16 were diverse papillary lesions, 36 areas were termed “visually suspicious for malignancy”, and 35 areas were relatively normal in appearance. OCT had an overall sensitivity of 100%, overall specificity of 89%, positive predictive value of 75%, and negative predictive value of 100%. Goh et al. [18] reported the results of 94 OCT images in 32 patients. Muscle-invasive bladder cancers were detected with 100% sensitivity and 90% specificity. The sensitivity and specificity for Ta lesions were 90% and 89%, respectively. However, the conventional OCT system is only two-dimensional imaging, and modification of image capture in 3D is much more time-consuming with current modalities.

Recently, our institution developed the ultra-high speed OCT system that enables real-time display of various 3D images without the limitation of diagnostic time. We had performed pre-clinical experiments of real-time OCT images in exenterated trachea and bladder using porcine model. Real-time OCT was shown precious layers like histologic finding on the images. According to these results, we firstly reported biological images of real-time OCT in human bladder and ureter. These 3 cases were examined from the lumen side of bladder or ureter. In addition, we can take images whole bladder and ureter area, including cancerous, normal and boundary areas. However, the imaging area by this new OCT system was demonstrated around 12 mm from the probe, and the depth is approximately 2 mm from the urothelium. In our experience, the perivesical fat layer was deeply located from the probe and cannot be observed using current system.

In this study we examined real-time 3D imaging by ultra-high speed OCT of exenterated advanced UC and compared the images with results from histopathological examination. First, the real-time 3D OCT images of normal bladder wall and ureter were confirmed three tissue layers, specifically the urothelium, lamina propria, and muscularis layer. Second, there were no structural layers in the muscle-invasive UC area. Third, the three characteristic layers were not found in the scar tissue area.

A limitation of this study was that real-time 3D imaging was done on an excised specimen and not a preoperative sample. We diagnosed these 3 cases as advanced UC preoperatively by the existing diagnostic modalities of CT or MRI. Therefore, simple comparisons of CT with OCT images were relatively difficult. In addition, all three patients were advanced UC cases, and there was neither a nonmuscle-invasive UC nor a CIS case. However, in real-time 3D OCT, it may be possible to immediately identify nonmuscle-invasive UC range, particularly for CIS. In a lesion suspicious for muscle-invasive UC, real-time 3D OCT may show precious diagnosis with no three-layer structures and may avoid the need for a second TUR.

## Conclusions

This study highlighted a new diagnostic method with potential application for UC diagnosis. We conducted real-time 3D imaging of advanced UC by ultra-high speed OCT. We will investigate additional cases and expect that cystoscopy or ureteroscopy with ultra-high speed OCT will be developed in the future.

### Consent

Written informed consent was obtained from the patient for publication of this case report and any accompanying images.

## Competing interests

The authors declare that they have no competing interests.

## Authors’ contributions

MI and KM contributed to conception, design, and drafting of the manuscript. MN and TF contributed in drafting the manuscript. DC and KO contributed to acquisition of real-time 3D imaging by OCT. KO, KS and MI contributed in drafting the manuscript and revising it critically for important intellectual content. All authors read and approved the final manuscript.

## Pre-publication history

The pre-publication history for this paper can be accessed here:

http://www.biomedcentral.com/1471-2490/13/65/prepub
